# Branding in the eye of the storm: the impact of brand ethical behavior on brand commitment during the COVID-19 crisis in a South American country

**DOI:** 10.1057/s41270-022-00188-1

**Published:** 2022-10-10

**Authors:** Jose Ribamar Siqueira Junior, Enrique ter Horst, German Molina, Laura H. Gunn, Felipe Reinoso-Carvalho, Burcu Sezen, Nathalie Peña-García

**Affiliations:** 1 Department of Business Administration, Pontificia Universidad Javeriana, Carrera 7 No. 40 – 62, Bogotá, Colombia; 2 School of Management, University of los Andes, Bogotá, Colombia; 3 Idalion Capital Group, USA, London, UK; 4 Department of Public Health Sciences & School of Data Science, University of North Carolina at Charlotte, Charlotte, NC 28223 USA; 5 Faculty of Medicine, School of Public Health, Imperial College London, London, UK; 6 Research Department, CESA, Bogotá, Colombia

**Keywords:** Brand ethicality, Turbulent times, COVID-19, Bayesian methodology, Brand commitment

## Abstract

The purpose of this study is to determine how consumer perceptions of brand ethical behavior can affect their commitment to brands during turbulent times. A study of the effects of perceived ethical behavior of brands in Colombia during the initial months of the COVID-19 outbreak was conducted in May 2020 in Bogota, Colombia, to ascertain customer perception of brand ethical actions during the first 2 months of the COVID-19 outbreak. A Bayesian model was developed to assess the impact of brands' ethical behavior on brand recognition benefits, brand image, and brand commitment. The selection of the initial months of the outbreak for this project was important because the COVID-19 pandemic had the potential to augment consumer perception of brands' ethical actions at a pivotal moment in consumers' lives. Our findings demonstrate that customers expressed a positive perception of brands' ethical actions during the early months of the pandemic, which resulted in high commitment intention to these brands in the model examined. The findings highlight the critical nature of the perception of ethical behavior in the eyes of customers during a major global health crisis. More than ever, organizations must commit their brands to fully live out their publicly expressed ethical principles and continuously monitor consumer perceptions of brand attributes and behaviors associated with ethical actions regardless of market conditions. Commitment to living the brand's stated ethical principles can be demonstrated via branding activities in ways that can be especially welcomed by customers during turbulent times. The insights mentioned in this article are crucial for brands already present in South America or exploring expansion into these regions. The findings provide compelling evidence of the impact of ethical actions on consumer commitment to brands, suggesting that brands must now, more than ever, stay in touch with their customers and truthfully live the ethical values they transmit to customers.

## Introduction

Businesses have realized they can no longer rely solely on traditional methods to sustain their market presence. The necessity to evolve, live out, and act on their guiding principles has been made evident by the COVID-19 pandemic's impact on customer behavior. Brands must analyze, absorb, and comprehend how current real-world experiences shape consumers' expectations. The pandemic has shown that even if a consumer is currently satisfied with a brand, the quest for future consumption options does not end (Trentacosta 2020) as consumers may continue to test new brands while expressing satisfaction with their present choices. This behavior holds true in times of crisis, as highlighted by a study published by McKinsey (2020), which showed that 40% of consumers admitted they had switched brands or retailers during the COVID-19 pandemic. This issue is exacerbated by customers' real-time access to brand information, user feedback and reviews expressing positive and negative evaluations of a brand (Lynch and Ariely 2000). Additionally, as a result of customer expectations of ethical brand conduct, consumers want their favorite brands to actively engage in corporate social responsibility (CSR) activities. Now, more than ever, a brand's ethical behavior can critically influence customers’ perceptions of the brand (Ferrell et al. 2019).

The brand building process suggests that firms can and should control brands. Brands, in the firm's view, are assets. As such, brands' qualities, functions, and duties should be assessed from a strategic and financial viewpoint (Swaminathan et al. 2020). An attribute, such as a brand's image, is dynamic and depends on actions that accumulate over time (Martínez and de Chernatony 2004). A brand's attributes must be exposed to consumers to affect their perception (Westcott 2001). All organizational behaviors that affect and drive brand commitment, including brand ethics, can achieve this. According to Singh et al. (2012, p. 543), consumers can regard brands based on attributes such as “honest, responsible, and accountable toward numerous stakeholders.” The impact of moral philosophy on ethical theory is reflected in a brand's perceived ethicality (Newholm et al. 2005). Brands can help firms build trust, stability, and distinctiveness among internal and external stakeholders (Kay 2006). Brand ethics is, therefore, crucial for organizations operating in turbulent conditions, like global crises.

To boost a brand's image, brands should build and present a true brand consciousness through consistent ethical practices (Iglesias and Ind 2016). Most existing research on ethics in marketing focuses on the impact of ethical or socially responsible practices on brand outcome variables, such as customer trust (Swaen and Chumpitaz 2008), and purchase intentions among other behaviors (Luchs et al. 2010). Swaminathan et al. (2020) recently underlined the necessity to recognize societal problems posed by brands associated with social responsibility, sustainability, and human resource practices. This shift in brand behavior should also consider the changing consumer perspective, which is now more embedded in society, with customers dictating societal trends and, hence, management practice, leading corporations to now treat “brand ethics” as a competitive advantage (Brunk 2012).

With the above being exposed, the purpose of the present study was to examine the research question of how consumer perception of brands ethical behavior during turbulent times can impact their commitment to brands. Departing from Iglesias et al. (2019) original focus on brands that operate in the services sector, this study’s first contribution is an examination of the effects of perceived ethical behavior of brands in general, unconstrained by a particular segment. The second contribution is the focus on the relationship between brand ethicality and brand commitment, which remained unexplored in the aforementioned study. The final contribution is the investigation of a proposed model in a scenario characterized by the COVID-19 pandemic. The COVID-19 context has the potential to magnify the importance of brand ethical behavior raising questions about its impact on customer perception of brand ethicality and ensuing brand commitment. During the COVID-19 crisis, consumers expressed a desire to know what brands and businesses were doing to protect their employees' health and jobs. More specifically, 73% wanted to know what businesses were doing to protect their customers from getting sick (Trentacosta 2020).

The salience of brand ethicality is anticipated to increase during crisis periods, such as recessions and the COVID-19 pandemic. This argument is based on the literature examining the effectiveness of marketing actions during recessions that suggest that economic fluctuations impact purchase decisions and brand considerations (Dekimpe and Deleersnyder 2018; Van Steenburg and Spears 2011). In particular, research suggests economic downturns force consumers to re-evaluate their brand choices (Raggio and Leone 2009). There are two reasons that support the idea that brand ethicality mattered more in COVID-19 times. First, consumers begin processing cues that suggest quality and value more deeply in unusual times (Quelch 2008), having increased risk perceptions and more difficulty making decisions (Dekimpe and Deleersnyder 2018). Therefore, consumers are more receptive to all signals given by the firm, including those of brand ethicality. Previous research on CSR has found that besides creating a positive halo effect on consumers’ brand perceptions, CSR also helps mitigate risk (Bhattacharya et al. 2021). Second, research shows that consumers will pay a price premium for a brand during recessions only when they are convinced of more outstanding quality (Steenkamp et al. 2010). However, what constitutes quality may be redetermined during these times of reconsideration of priorities and the general feeling of solidarity within society. Recent research demonstrates that firms’ CSR activities add to brand value more during recessions by impacting brand quality more strongly at these times (Bhattacharya et al. 2020). Hence, crisis situations may lead to consumers’ changing the weight they place on brand ethicality in their consumption decisions and therefore increase the perceived value of brands with ethical claims. The COVID-19 pandemic represented a turbulent scenario where brands could help firms create trust, stability, and differentiation among internal and external stakeholders (Kay 2006), while showing to customers that they do more than just “talk the talk.” Brands must now more than ever stay in touch with consumers.

To address the proposed research question, a survey was conducted in May 2020 to assess consumer perceptions of brand initiatives in Bogota, Colombia during the early stages of the COVID-19 epidemic. In an ideal scenario, the data for this study would be collected from the same cross-section before and during COVID-19 to provide contrast. As this was not possible, a point of comparison was established based on previous studies conducted prior to the pandemic and examining the impact of brand ethicality on consumer behavior through customer loyalty (Markovic et al. 2018) and brand equity (Iglesias et al. 2019) under normal market circumstances. The paper is organized as follows: first, an overview of the business impact of COVID-19 is presented and followed by a discussion about the proposed hypotheses development and theoretical model. Then the methodology used is discussed and followed by a presentation and analysis of results. The article concludes with a discussion about theoretical contributions, managerial implications, limitations and future research.

## COVID-19 research context

### The COVID-19 global scenario

The COVID-19 crisis is a systemic shock to the global economy that has had a similar impact on organizations operating in developed and emerging economies. At the time of writing, it is impossible to predict the long-term impact on the global economy with any certainty. Nonetheless, due to the interconnected nature of the global economy, some parallels can be drawn when examining the narrative about the impact of COVID-19 on organizations and the international business community so far, which can be found in the narrative about the impact of COVID-19 on the global economy. Because of the widespread use of lockdown and social distancing measures around the world, the short-term consequences of COVID-19 are felt almost immediately and effortlessly (He and Harris 2020).

Following the early restrictions and fluctuations in demand due to the pandemic, internet retailers benefited at the expense of smaller firms, causing asymmetric effects in commercial activity (Bartik et al. 2020). Companies that could adapt their production and distribution models survived, while others had to close temporarily or permanently (Pantano et al. 2020). While material and input costs were essentially unchanged, revenue expectations and the ability to fulfill on contracts were significantly impacted (NDA 2020). Sharp income declines impacted not only staff retention, but also debt servicing, which in certain countries was eased by emergency government credit programs (U.S.C.C. 2020). Industries had to quickly adapt to new needs. For example, some producers of alcohol-based items shifted to hand sanitizers, and apparel makers shifted to face masks (Roggeveen and Sethuraman 2020). While economies with producers focused on some basic products saw extraordinary demand, regions and countries relying on tourism and transportation sectors were disproportionately hit (Uğur and Akbıyık 2020).

Inequities grew deeper with the pandemic. For example, in the USA, female and minority-owned businesses were more likely to fail, leading to speculation that a similar trend was observed in Latin America (Fairlie 2020; Kawachi 2020), and although unexplored to date, it is likely that similar or worse inequities happened in developing regions such as Latin America. There was also increased income segregation (Bonaccorsi et al. 2020; Kawachi 2020). Decreased access to basic preventive treatment further exacerbated the disease-specific health impact of COVID-19 on communities (Horn and Haas 2020).

Even though the shape of the new normal is still unpredictable nearly 2 years into the pandemic, the impact of COVID-19 on individuals and enterprises is already measurable. The extent to which observed shifts in demand and increases in online retailing persist will likely impact the economy in the coming years (Roggeveen and Sethuraman 2020). Some companies, though, will be too financially vulnerable to survive (Bartik et al. 2020).

It has been argued that now is the time to transform the economic paradigm to more sustainable forms (Tokic 2020). These changes in the economic paradigm may result in shifts in marketing efforts from consumer materialism to consumer spiritualism (Mehta et al. 2020). The pandemic may increase the focus on sustainable consumerism. Consumers’ experiences are ‘outsourced’ to how society views those choices, which may lead to less autonomous decisions (He et al. 2012). A shift from well-having to well-being (Gardels 2000) and mindful spending is already being valued in global financial markets. Environmental, Social, and Governance (ESG) criteria are becoming more relevant (Broughton and Sardon 2020), and ESG-based funds have had record inflows even before the epidemic (Iacurci 2020), with no indication that the trend is spurious. This time the pressure toward social responsibility may come from both investors and consumers. Due to the asymmetric knowledge that consumers face, firms may have previously focused on marketing to primarily uneducated consumers rather than moving toward accountable change. Informed-shareholder pressure (Bae et al. 2021; UBS) has recently become a more effective trigger for change (Testa et al. 2018).

The impact toward ESG focus in the global financial markets may help translate brand reputation into equity (Mahmood and Bashir 2020). The pandemic and its aftermath may offer a unique opportunity for firms to transform corporate marketing efforts into meaningful corporate change as a means to differentiation and access to increasing informed consumers and ESG-favoring financial opportunities (He and Harris 2020).

### The COVID-19 Colombian scenario

South America was selected for the study because very little marketing research based on data gathered in South and Latin America has been published in high quality business and marketing academic journals in the past (Fastoso and Whitelock 2011). The writing this article took place during the first period of lockdown in Colombia when the implementation of social distancing measures was in full effect. Colombia was selected for this research project due to its strong economic performance relative to other countries in South America in 2019, the year prior to the start of the Pandemic. The selection was based on overall GDP performance of the top economies in the region in the year 2019: Argentina (− 2.0%), Bolivia (− 2.2%), Brazil (1.4%), Chile (0.9%), Colombia (3.3%), Ecuador (0.0%), Paraguay (-0.4%), Peru (2.2%) and Uruguay (0.4%) (World Bank 2022). A number of significant factors made Colombia an ideal and timely setting in which to examine the corporate ethical actions taken by various brands in response to the pandemic, particularly at its early stages. First and foremost, the period for the initial outbreak in Colombia is clearly delimited at the time of the study. According to MinSalud (2020), the initial outbreak in Colombia can be tracked to the first positive COVID-19 case identified on March 6 of 2020, leading to the declaration of health emergency by local authorities on March 12, 2020 (Reuters 2020) and closure of borders on March 16, 2020 (Tiempo 2020). Second, because there were no significant external events occurring at the same time, it was possible to conduct a clear examination of consumer perception of brand actions during a critical stage of the outbreak that was marked by high uncertainty. Colombia's situation was very similar to that of other countries, which had had more time to prepare and learn from China's experience dealing with the pandemic in the past. Finally, similar to what happened in other countries and China (Chen et al. 2021), the COVID-19-related policies implemented in Colombia were unified and applied throughout the country, allowing the consequences of the pandemic to be comparable across all of the country's departments and regions (Republica 2020).

## Hypotheses development

### Brand commitment

Consumers' ethical judgments of brand conduct are becoming increasingly crucial, influencing purchasing decisions and brand loyalty. Consumers' increasing emphasis on brand ethical behavior has been attributed to increased ethical consumerism. Consumers have become substantially more aware of brand behavior (Sudbury-Riley and Kohlbacher 2016). As a result, any brand ethical breach now impacts consumer perception and the subsequent relationship with the brand (Brunk and Blümelhuber 2011). According to Das et al. (2019), negative impressions of brand ethical behaviors, or brand ethicality, can add a moral dimension to the feeling directed at a brand, thereby reducing brand commitment. Conversely, positive brand ethical conduct impressions might motivate customers internally, leading to improved brand commitment (Vallerand et al. 2003).

The literature on brand commitment also examines its relationship with brand loyalty. Consumers who are highly committed to a brand may show a strong active interest in product or brand knowledge, rather than considering switching brands (Warrington and Shim 2000). Nonetheless, a consumer's current satisfaction with a brand should not be interpreted as a sign of brand loyalty or as a sufficient reason to avoid abandonment of the brand during future consumption decisions. Although brand loyalty and brand commitment are theoretically linked, they differ in key important ways. Lastovicka and Gardner (1979) defined brand commitment as an emotional or psychological relationship to a brand that shows consumer irreplaceability intention. While brand loyalty occurs when the existence of brand commitment is identified, the opposite does not hold since brand loyalty might be a reflection of a different need that does not necessarily express the irreplaceability of a brand in the mind of the consumer (Mitchell 1998). Other elements associated with brand commitment include relationship investment, relationship termination costs, communication, shared values, and involvement (Morgan and Hunt 1994; Sargeant and Lee 2004). Involvement has been a key marketing topic for the last 50 years, with roots identified in early research on television advertising (Warrington and Shim 2000). Product involvement was differentiated from brand-decision involvement and ego participation by Fornell and Larcker (1981), who hypothesized the aforementioned distinctions using statistical tests for “differentiation in conceptions.” Warrington and Shim (2000) relate the growth of participation to three elements: (1) individual traits; (2) situational aspects; and (3) article or stimulus qualities. This is important because high levels of involvement lead to increased time and effort spent on searches (Bloch 1986), longer decision-making periods, increased perception of product features, along with the potential to impact brand preference (Zaichkowsky 1994).

Customers tend to build a stronger affection for organizations that share cognitive identities with them (Bhattacharya and Sen 2003). When a brand operates in a way that is inconsistent with its previous ethical behavior, it might cause consumers to feel conflicted. It forces a customer to examine their own identity and how peers perceive their new conduct (Das et al. 2019). This ethical breach can result in brand rejection, boycotts, retaliation, and unfavorable word-of-mouth (Shaw et al. 2006).

### Brand ethicality

Ethicality is grounded on two streams of ethical theories: deontology and teleology (Forsyth 1992; Frankena 1973; Newholm et al. 2005). To judge if an activity is morally right, or not (i.e., immoral), deontology contends that a set of rules should be founded on higher moral standards or the law. The deontological perspective requires corporations to act legally and follow current laws, where the moral evaluations of consumers are based on honesty, integrity, and other moral principles (Shanahan and Hyman 2003).

#### What determines ethicality? A brief overview of moral philosophy

Generally, the concept of ethics refers to moral rules or principles that guide people's actions (Sherwin 1983). Moral judgment helps individual classify actions as right or wrong. Moral concepts might be neutral or ambivalent. There are two main ethical schools of thought: deontology and teleology (Forsyth 1992; Frankena 1973; Newholm et al. 2005). They differ on consequentialism, which refers to judging morality by rules or social consequences.

Deontology is a non-consequentialist theory. This school's most notable contributor is Immanuel Kant. An individual would assess the morality of a course of action by consulting higher moral responsibility, norms, or the law. An action's ethicality is determined by the decision-maker's values. This ethical school of thought establishes universal principles of right and wrong, but ignores the social repercussions of actions based on these norms. From a corporate perspective and according to Clement (2006), the morality of corporate activity is regulated by a legal framework (i.e., companies are unethical only to the extent that they violate the law).

Teleology is consequentialist (i.e., it judges actions on the basis of their outcomes, and when compared to outcomes from alternative actions). This perspective allows for the influence of perceived consequences, their probability of becoming a reality, their desirability, and the severity of the outcome—positive or negative—in making final decisions regarding morality. However, since people can rationalize any alternative to an outcome if they focus on whether the intended result is maximizing the benefit gained, and there are different forms of teleological moral philosophy prioritizing the sake of different parties—the individual or the society, the literature provides various explanations as to how to approach to the balance between benefit and harm (Derry and Green 1989; Hunt and Vitell 1986; Whetstone 2001). For example, from a consequentialist marketing manager's perspective, the ethical decision has been found to be primarily associated with actions leading to the desired financial performance (Dyck and Manchanda 2021). Likewise, application of fear appeals in marketing communications of health-related products for elderly people can be considered as a reasonable advertising approach, which may enhance the consumer’s quality of life when other alternatives seem less effective (Benet et al. 1993).

Utilitarianism is the most prominent theory within teleologism, and is connected with Jeremy Bentham and John Stuart Mill (Bentham 1996; Mill and Belot 1897). The utilitarian theory argues that the decision-maker is obliged to seek the optimal outcome considering all parties affected by it. Therefore, in contrast to deontology, utilitarianism is concerned with the broad impact on society rather than emphasizing the individual (Crane and Matten 2007).

#### How do consumers perceive ethicality?

While these two ethical normative theories outlined above are stated as mutually exclusive (i.e., scholars either subscribe to a consequentialist or non-consequentialist position to determine what is ethical) (Beauchamp and Bowie 1979; Derry and Green 1989), the virtue ethics approach, which creates a balance between the two opposite extremes of deontology and teleology, was another moral philosophy type that also received scholars' attention (Macdonald and Beck-Dudley 1994). From the marketing management point of view, the question arises as to whether consumers judge ethicality using consequentialist or non-consequentialist decision-making criteria? However, the question remains on how consumers judge ethicality, using consequentialist or non-consequentialist decision-making criteria? Some scholars have suggested that individuals use a combination of deontological and teleological norms in their moral decision-making (Shanahan and Hyman 2003; Vitell et al. 2001).

Brunk (2012) investigated this research question by introducing the consumer perceived ethicality (CPE) scale. The author discovered that customers combine the two schools' ethical ideals. The six themes derived from consumer interviews revealed a mix of deontological (obeying the law, as well as morality) and teleological (societal responsibility, avoiding harmful action, and evaluating good and bad outcomes) rules.

#### Brand ethicality as defined in the academic literature

There are multiple different definitions of what brand ethicality actually means. A common factor across definitions is that ethical brands are expected to contribute to the well-being of society, or the consumer, by fulfilling the needs and desires of all stakeholders (e.g., through organic ingredients, fair trade, etc.) (Ferrell et al. 2019).

While delineating brand ethicality, Ferrell et al. (2019) argued that it is crucial to differentiate it from CSR initiatives. The academic literature has primarily used such two terms interchangeably (Fassin et al. 2011), whereas practitioners see the two constructs as clearly distinct (Weller 2017). Ferrell et al. (2019) has shown that CSR and business ethics have different outcomes, with ethics contributing more to changes in brand attitude than CSR. Despite the importance of this distinction, it has been shown that: (1) consumers see both concepts as equally important when asked directly (Ferrell et al. 2019); and (2) when consumers describe company/brand ethicality, they see both elements of CSR and business ethics as relevant to their definition (Brunk 2012).

In brief, brand ethicality has been defined as social responsibility, which combines corporate social performance, stakeholder theory, and business ethics theory (Carroll 1999). Thus, as this study takes a consumer perspective, we use Brunk (2012)'s scale, which adopts items that apply simultaneously to CSR and business ethics to measure brand ethicality.

#### Consequences of brand ethicality

Kumar and Reinartz (2016) argued that brand ethicality is a vital antecedent to brand attitudes. However, results in the literature are mixed while assessing the impact of ethical brand perceptions on consumer behavior. For instance, Peloza et al. (2013) found that product performance is overwhelmingly more important than ethical product attributes. On the other hand, Singh et al. (2012) found that the perceived ethicality of a brand is related to brand loyalty through the mediation of brand trust. Therefore, the present study is meant to contribute to the needs for further research in this area. Based on the relationships between brand ethicality with brand commitment previously examined, we posit that:

##### Hypothesis 1

Brand ethicality will have a significant impact on brand commitment.

### Brand recognition benefits (BRB)

BRBs refer to the feeling of privilege that customers feel while adopting and/or using a brand, when compared to customers of other brands (Shugan 2005). Therefore, recognition benefits principally consider a customer's positive emotions, feelings, and affect for a specific brand (Wagner et al. 2009). The mechanism through which recognition benefits operate is usually theoretically explained as boosting customer status, inducing them to feel more successful than others, and that others perceive such customers as somehow superior (Wagner et al. 2009). Such boost in self-esteem can make customers more loyal to brands and, thus, result in higher brand equity.

Within our context of the rising importance of ethical consumerism (Carrigan and Attalla 2001; Shaw and Shiu 2002), and especially upon the dynamics in consumer behavior triggered by the COVID-19 crisis, it is likely that brands who actively and publicly carry out ethically correct actions would induce consumers to reach their self-actualization goals at higher levels and, therefore, feel more satisfied with their brand choice (He and Li 2011; Martínez and Rodríguez del Bosque 2013). Despite the lack of research concerning this important construct, recognition benefits may be an important antecedent of brand evaluation.

#### Hypothesis 2

Brand ethicality will have a significant impact on BRBs.

#### Hypothesis 3

BRBs will have a significant impact on brand commitment.

### Brand image

A company's brand can be one of its most valuable assets. As a result, firms must guard against actions that erode a brand's value (Martínez and Pina 2010). Although brand image research was found to date back to the early 1950s (Merz et al. 2009), there is no universally accepted conceptualization or measurement for it (Hsieh and Li 2008; Park and Rabolt 2009). Nonetheless, most experts agree that a brand's image is formed by customers' impressions of that brand (Anselmsson et al. 2014; Cho and Fiore 2015).

A brand's image is shaped by its personality and familiarity, as identified by Kaur and Kaur (2019). Consumers' choice of a brand image as a favorite increases the brand's influence (Anselmsson et al. 2014; Cho and Fiore 2015). Brand image can be used as an external cue to help people judge product quality. Consumers link brands portraying powerful images with greater quality, which can operate as a surrogate for product or service guarantee (Lee et al. 2011), showed that a brand with a good image can make consumers think its items have good quality.

A systematic review from Plumeyer et al. (2019), showed that most articles about brand image did not define it. They attributed this to the possibility that these articles were not trying to measure brand image. Among those that provided a definition, the ones mostly adopted were either Aaker (1991) or Keller (1993) conceptualizations of brand image from their seminal works on brand management. Aaker (1991) described brand image as a collection of relevant associations that make up a brand image and are often reflected by product qualities, consumer advantages, or relative price. Keller (1993) later expanded the topic regarding brand image by substituting “associations” with “perceptions.” These associations should be able to recall the brand's meaning. Brand associations are concrete and intangible (Barreda et al. 2020).

Brands have been shown to influence social trends, act as catalysts for social interaction, and as societal symbols (Holt 2002). Brands are also becoming more socially active by aligning themselves with social and political issues. In this situation, purpose-driven brands must uphold societal values to empower consumers who use them to support social causes (Swaminathan et al. 2020). Brands strive to remain relevant in consumers' eyes by taking a stand on social issues, but there is no clear formula. Nonetheless, brands now have the potential to drive social change and are more closely linked to society's future (Clifton 2009). This also benefits from the ability to amplify a brand's social message through hyperconnectivity. Customers' expectations of ethical behavior may therefore increase as a result of new hyper-connected environments (Iglesias et al. 2017; Shaw and Shiu 2002). Brand identities must not only incorporate ethics (Iglesias and Ind 2016), but also convey this commitment to customers (Rindell et al. 2011).

Ethical branding combines business ethics and brand management (Fan 2005). Ethical brands should not only avoid harm (Williams and Aitken 2011) but must also strive to be ethical (Fan 2005; Story and Hess 2010). This unprecedented level of hyperconnectivity has given customers unprecedented access to brand behavior data. As a result, consumers expect brands to demonstrate CSR (Balmer 2001) and to live up to their promises (Maxfield 2008). Consumers' ethical concerns led to the term “consumer perceived ethicality” being coined by Brunk (2010). The author argues that consumers tend to perceive brands as ethical if they respect moral norms, laws, and society, among other issues. The development of a strong brand equity requires that brands recognize and nurture an environmentally responsible behavior, according to Reverte (2012). Ethical branding combines business ethics and brand management (Fan 2005). Ethical brands not only should avoid harm (Williams and Aitken 2011) but must also strive to be ethical (Fan 2005; Story and Hess 2010). This unprecedented level of connectivity has given customers unprecedented access to brand behavior data. As a result, consumers expect brands to demonstrate CSR (Balmer 2001) and to live up to their promises (Maxfield 2008). Consumers' ethical concerns led to the term “consumer perceived ethicality” being coined by Brunk (2010). He argues that consumers tend to perceive brands as ethical if they respect moral norms, laws, and society, among other issues. The development of a strong brand equity requires that brands recognize and nurture an environmentally responsible behavior, according to (Reverte 2012). Customer loyalty and perceived service quality (Mandhachitara and Poolthong 2011), customer affective commitment (Chomvilailuk and Butcher 2014), customer satisfaction (He and Li 2011) and recognition benefits (Khan et al. 2015) have all been studied previously. The importance of ethics for brands is further highlighted in the case of brands whose reputations have been blemished by a crisis and need to invest in ethicality to improve brand image (Iglesias et al. 2019). Additionally, the purchase of a product or service of an ethical brand can endow customers with recognition benefits. In accordance with this rationale, we hypothesize that:

#### Hypothesis 4

BRBs will have a significant impact on brand image.

#### Hypothesis 5

Brand ethicality will have a significant impact on brand image.

#### Hypothesis 6

Brand image will have a significant impact on brand commitment.

## The conceptual framework

According to Ferrell et al. (2019), when customers evaluate a brand's ethical performance, they consider the brand's impact on their well-being through the fulfillment of their wants and desires. When firms address the customer’s requirements and desires, the customer experience may be favorably evaluated by its patrons, resulting in high levels of satisfaction (Oliver 2014). Historically, evidence has been found of a sequential relationship between consumer happiness and loyalty (Peña-García et al. 2018). The purpose of this article is to fill a void in the literature on consumer brand commitment, which is a higher-level concept than loyalty. As a result, H1 implies that perceived brand ethicality has a considerable effect on brand commitment.

This study proposes the inclusion of BRB in the research model to conform with the original model proposed by Iglesias et al. (2019) which served as the basis for this study. Brand commitment is a result of the consumer's perception of privilege connected with a brand in comparison to other brands' customers (Shugan 2005). Because perceived brand ethicality can be a competitive advantage in brand building (Brunk 2012), it can be assumed that there is a difference in consumer perception between brands with explicit ethical principles versus those without those ethical principles. Thus, for clients who review a brand's ethical behavior, it should result in a larger sense of BRB than for others, as theorized in H2. Similarly, H3 proposes that customers with a favorable BRB perception will display a higher level of brand involvement. H4 asserts that the brand's ability to provide a sense of well-being and raise consumers’ self-esteem has an effect on the brand's image, as customers attribute a higher value to the brand as a result of their favorable sentiments toward it.

**Fig. 1:**
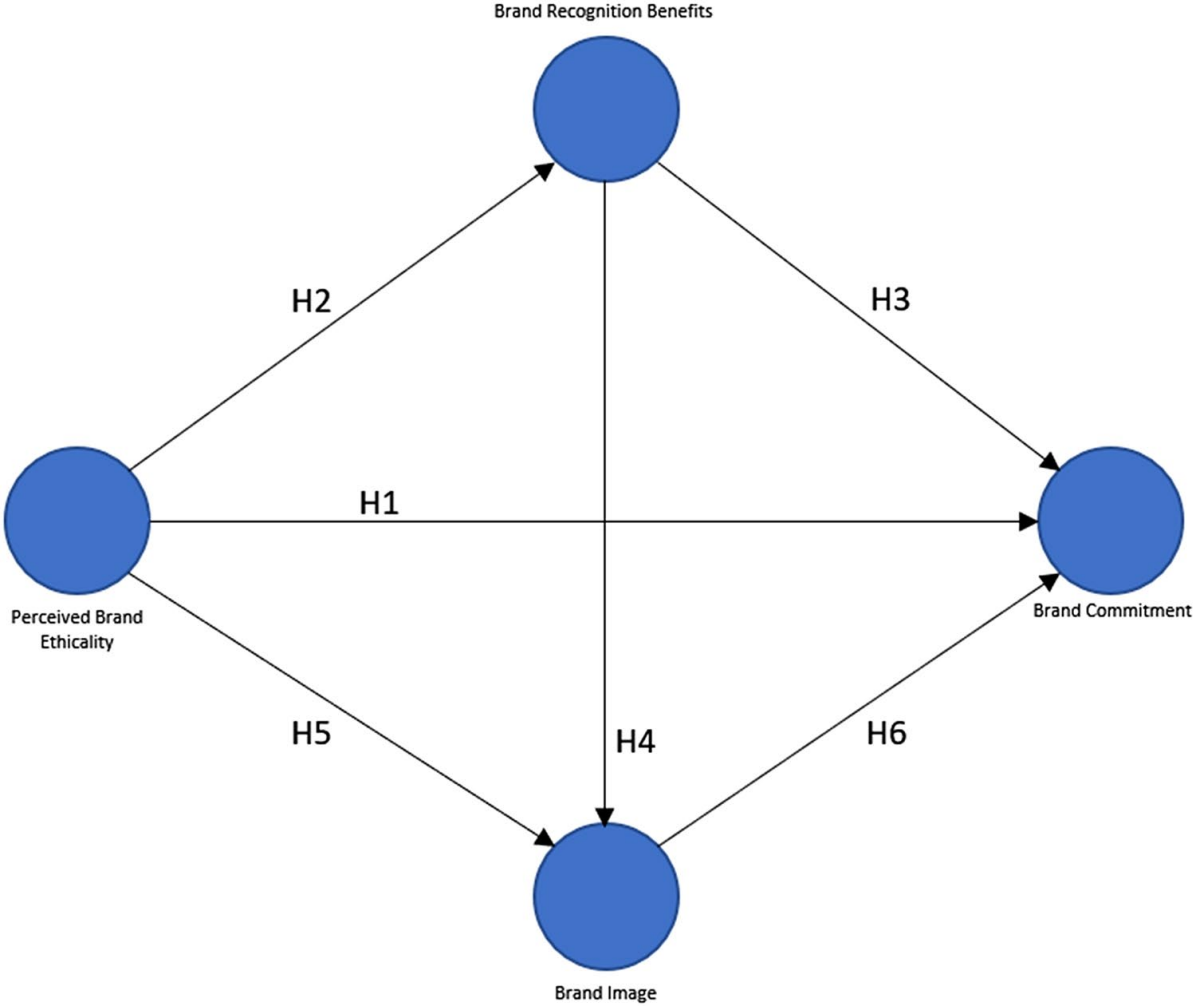
Proposed research model

According to Anselmsson et al. (2014), a brand's image is formed by consumer perceptions of it. As a result, H5 is proposed in this study, which examines the relationship between perceived brand ethicality and brand image, with the understanding that perceived ethics contributes to the brand's high concept by providing the consumer with a sense of differentiation, uniqueness, and emotional benefits. Similarly, H6 indicates that a strong brand image may have a favorable effect on consumer brand commitment, assuming that emotional advantages are a critical competitive advantage in the establishment of consumer brand commitment. The proposed research model is presented in Fig. 1.

## Methodology

### Sample

Recruitment of research subjects might be challenging when studying perception in turbulent times, such as the COVID-19 pandemic. External pressures might further complicate the data collection procedure. These pressures, manifested in the COVID-19 pandemic, presented both obstacles and opportunities within this investigation. The COVID-19 outbreak forced most governments to establish population mobility restrictions. One of the main motivations for this study was the belief that these governmental actions could trigger major changes in consumer habits and increased internet activity. With social distancing measures in place, customers had to turn to the internet for social interaction. Social networks became vital during this period of isolation, providing a unique setting for social research. Therefore, we opted to collect data for this study online. One of the key difficulties with using the internet as a venue for recruitment is the quality and response rate during the data collection stage and the risks of selection bias (Baltar and Brunet 2012). Some authors argue that the response rate of online studies is correlated to several factors such as the implementation of personalized contact strategies (Cook et al. 2000), the degree of interest of the individuals on the topic being researched (Groves et al. 2004) and the types of incentives offered (Couper 2000). The survey prepared for this study aimed to consider all those factors.

A snowball sampling approach was selected to leverage familiarity with the survey sharer, allowing for a more personalized survey receiver interaction. Snowball sampling allows current study participants to attract new study participants from their social networks. This sampling strategy is appropriate for hard-to-reach populations. Additionally, the selection of a current topic that had a substantial impact on their life motivated them to participate in the study.

The survey was kept as short as possible without surrendering any crucial items from the questionnaire, and all individuals who agreed to actively participate were offered the possibility of participating in raffle upon completion. Completeness and engagement were assessed afterwards and incomplete responses were discarded. The poll was developed online using Google Forms and the specificity of the instructions programmed for each question accounted for the following factors: (1) the visibility of questions on a window; (2) the ability to choose an answer with a single click on the desired option; and (3) the ability to apply filter questions that can select the suitable questions that participants must complete, as discussed in Baltar and Brunet (2012).

Consumers in Colombia's capital were polled and a self-identification filter was used to ensure participation of only Colombian nationals and legal residents. The questionnaire link was initially delivered via email or WhatsApp to undergraduate and graduate students at three universities in Bogota, Colombia, as well as to one of these schools' alumni database. After completing the poll, participants could share the link with direct connections via instant messaging or social media. The survey included information about the study's principal objective, voluntary participation, and data confidentiality in a brief introduction that also contextualized the survey. The survey's preamble emphasized that the questions were designed to examine participants' perceptions exclusively of how familiar brands (favorite brands, admired brands or simply brands used regularly in their households) responded to the COVID-19 pandemic. Consumers were not asked to name specific actions undertaken by the brands selected, but rather what their perceptions of brand actions were.

From May to June 2020, 785 surveys were completed, 24 of which were discarded owing to participant disengagement (no significant standard deviation in answers), leaving a final sample of 761 valid questionnaires. The sample was well-balanced, with 44% men and 56% women. The full composition of the sample can be found in Table 1.

**Table 1:** Sociodemographic traits

Variable	Category	% Responded
Sex	Male	44
	Female	56
Age	18–25	22
	26–35	31
	36–45	28
	46 and over	19
Marital status	Single	43
	Married	42
	Divorced	6
	Widower	1
	Other	8
Education	High school	11
	Some high school	1
	Professional certificate	22
	Graduate degree	16
	Undergraduate degree	51
Monthly income	Less than $1,000,000	11
	$1,000,000–$2,000,000	16
	$2,000,001–$4,000,000	21
	$4,000,001–$11,000,000	31
	Over $11,000,000	21
Occupation	Unemployed	17
	Employed	54
	Student	13
	Self-employed	16

All monetary figures are presented in Colombian Pesos

### Latent variables

See Table 2.

**Table 2:** Measurement Items

Latent variables	Measurement items	Item	References
Perceived Brand Ethicality	This brand respects moral laws This brand always adheres to the law This brand is socially responsible This brand avoids damaging behavior at all cost This brand is a good brand This brand will make a decision only after careful consideration of the potential positive or negative consequences for all those involved This brand is concerned to improve the well-being of society This brand follows high ethical standards	PBE1 PBE2 PBE3 PBE4 PBE5 PBE6 PBE7 PBE8	Brunk (2012) Salmones et al. (2005)
Brand Recognition Benefits	Being a customer of this brand makes me feel privileged compared to others Being a customer of this brand makes me feel special compared to others Because I am customer of this brand others look up to me Being customer of this brand makes me demonstrate greater success than others	BRB1 BRB2 BRB3 BRB4	Wagner et al. (2009)
Brand Image	This brand provides good value for money There is a reason to buy the brand instead of others The brand has personality The brand is interesting I have a clear impression of the type of people who consume the brand This brand is different from competing brands	BIMG1 BIMG2 BIMG3 BIMG4 BIMG5 BIMG6	Martínez and de Chernatony (2004)
Brand Commitment	I do not feel “emotionally attached” to this brand (reversed) This brand has a great deal of personal meaning for me I do feel a strong sense of belonging with this brand It would be very hard for me to leave this brand right now, even if I wanted to Too much in my life would be disrupted if I decided I wanted to leave this brand now Right now, staying with this brand is a matter of necessity as much as desire If I had the opportunity to shop with a better provider elsewhere, I would not feel it was right to leave Even if it would be to my advantage, I do not feel it would be right to leave this brand I would not leave this brand right now because I have a sense of obligation to them This brand deserves my loyalty I would feel guilty if I left this brand now	BC1 BC2 BC3 BC4 BC5 BC6 BC7 BC8 BC9 BC10 BC11	Shukla et al. (2016)

### Measures

The data for this study were collected through a survey instrument developed using the multi-stage approach suggested by Morgan-Thomas and Veloutsou (2013). A panel of academic peers reviewed its content and face validity, finding it relevant to the study's setting and sufficient to specify each of the researched constructs. Each item was used as originally developed and all questions were double back translated by native speakers within a framework of collaborative and iterative translation following Douglas and Craig (2007) collaborative and iterative translation framework. The final measurement instrument was evaluated with two samples of 20 respondents each to examine instructions, response format, and measurement item clarity. Table 2 outlines the measures utilized in this investigation. Each item was measured using a 7-point Likert scale, ranging from “strongly disagree” (1) to “strongly agree” (7).

## Analysis and results

### Measurement model and description

We propose a Bayesian (hierarchical) Multivariate Ordered Probit model (Johnson and Albert 2006), with the hierarchical layers assessing the hypotheses of the manuscript. Each respondent (for each item) has been modeled independently, though coming from some unknown population of interest. Measurement items follow individual-specific Multivariate Ordered Probit structures, to accommodate the 7-scale Likert-based ordered nature of the data, and those items are associated with each of the four brand characteristics (latent constructs). An ordered categorical model is preferred over more traditional approaches that assume a natural equidistance between Likert scales (by considering Likert numerical responses as numerical variables), as there is no evidence of such equidistance within this survey. Additionally, even if designed toward such equidistance between scales, there is no guarantee that participants responded considering such equidistance. The observed categorical responses (scales) are, instead, mapped through data driven flexible thresholds into the corresponding latent constructs. The case of equi-distance is a possible outcome of the model, but not a constraint within the model. More details are provided in the “Appendix”. The relationships between those latent constructs, at the population level and through population-based hyper-parameters, are the quantities of interest. Each latent construct, which are unobservable measures of each of the four brand characteristics, is composed of a population-level mean and the sum of hypothesized individual-specific associations with the other brand characteristics. A more detailed description of the statistical components of the model can be found in “Appendix”.

Some of the advantages of the Bayesian framework include: (1) It is not reliant on large-sample assumptions (the posterior distribution accounts for the uncertainty in the data regarding the parameters of interest, including uncertainty related to potentially low sample sizes); (2) It is flexible, when needed, to adjust to missing data in a coherent, unified approach (Gelman and Meng 2004), though this was unnecessary in our study, where all responses contained complete information; (3) Model outcomes (posterior distributions of the parameters of interest) can be interpreted directly as unknown, random relationships between the constructs subject to uncertainty, instead of frequentist estimators with large-sample properties about those parameters (Winkler 2003); and (4) The proposed approach has been successfully demonstrated in the marketing literature (Dakduk et al. 2017; Reinoso-Carvalho et al. 2020; Siqueira et al. 2019, 2020). Additionally, prior information can be incorporated into the analysis, when available from previous studies (Winkler 2003).

Conclusions will be extracted from the posterior distributions, with evidence levels defined based on whether 99% of the mass of the posterior density lie in positive territory (very strong evidence), at least 95% of the mass lie in positive territory (strong evidence), or at least 90% of the mass lie in positive territory (moderate evidence). Any other case will be reported as no evidence. These are just subjective thresholds to map differing strengths of evidence to categories of that strength. The full 95% credible intervals (and, in fact, the full posterior distribution) provides the complete information about the strength of evidence about each hypothesis from this analysis.

### Results

A summary of the results is provided in Table 3. This table contains the posterior means and standard deviations for the key model parameters which relate to the hypotheses, as well as 95% credible intervals. It also provides a summary assessment of the evidence level based on thresholds described in the previous section. Although the Markov Chain Monte Carlo (MCMC) analysis provides the full distribution of the relationship parameters, we only report summary statistics of that posterior distribution.

**Table 3:** Model results, including posterior means and standard deviations, as well as central 95% credible intervals for the parameters (*β*) representing the association between constructs (hypotheses)

Hypotheses		Posterior mean (SD)	95% Credible interval	Evidence level
H1	Brand Ethicality → Brand Commitment	0.057 (0.042)	− 0.025, 0.141	Moderate
H2	Brand Ethicality → Brand Recognition Benefits	0.371 (0.036)	0.301, 0.441	Very strong
H3	Brand Recognition Benefits → Brand Commitment	0.443 (0.052)	0.342, 0.547	Very strong
H4	Brand Recognition Benefits → Brand Image	0.392 (0.052)	0.291, 0.495	Very strong
H5	Brand Ethicality → Brand Image	0.397 (0.040)	0.319, 0.476	Very strong
H6	Brand Image → Brand Commitment	0.166 (0.053)	0.062, 0.270	Very strong

Evidence level is defined based on the amount of posterior mass in the positive territory (> 99% very strong, > 95% strong, > 90% moderate)

For example, relationship H3, expressed through a linear relationship between latent constructs (*β*_3_), shows a posterior mean of 0.443 with posterior standard deviation of 0.052. The overwhelming majority of the posterior mass lies in positive territory, indicating strong evidence of the hypothesis. All parameters relating to the hypotheses demonstrate high amounts of mass in the positive territory. The only exception is H1, where the evidence is more moderate.

**Fig. 2:**
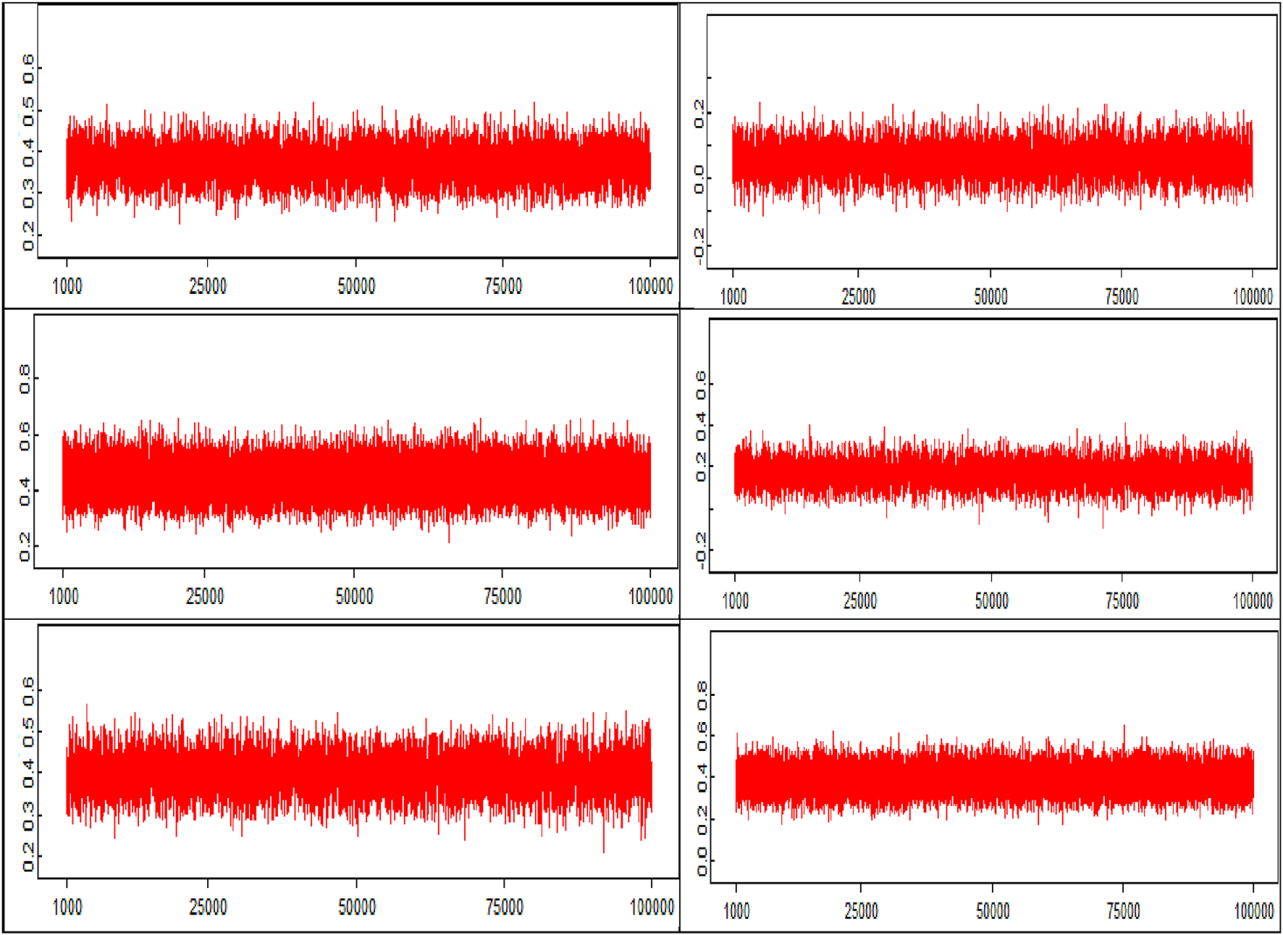
Traceplots for each of the six hypotheses (parameters *β*), from left to right and top to bottom

**Fig. 3:**
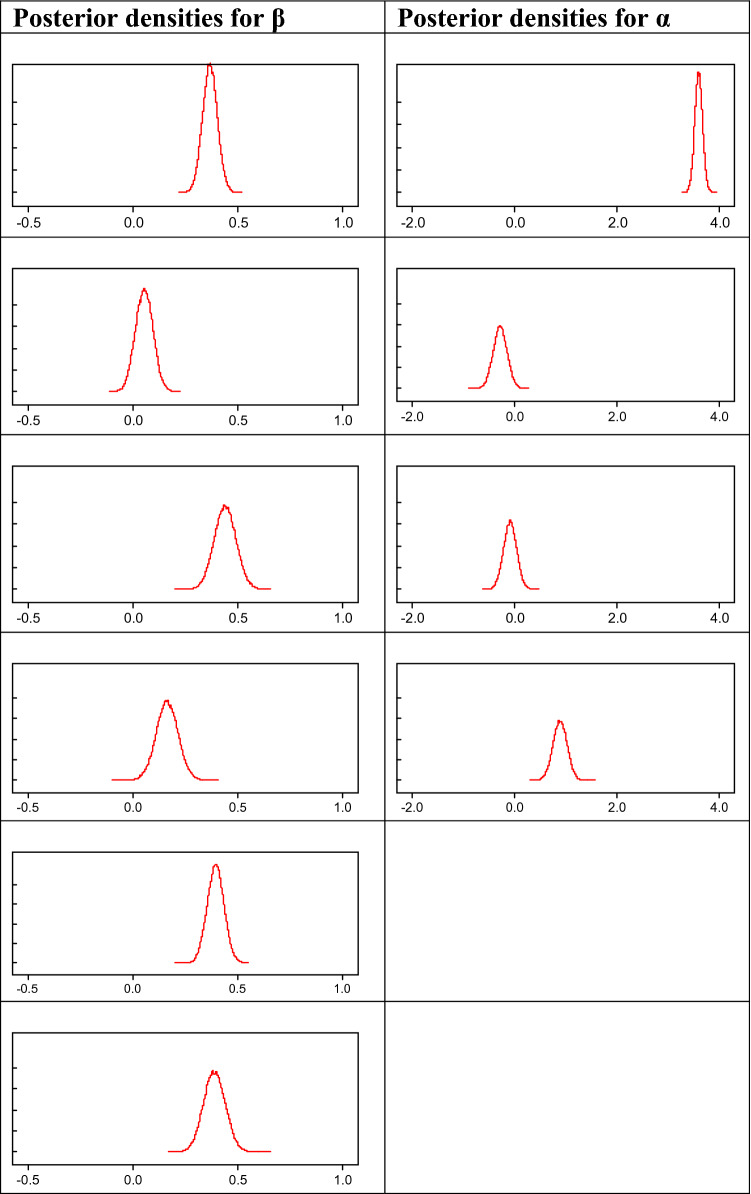
Posterior densities for the parameters *β* (left) and *α* (right). Parameters sorted from top to bottom (1–6 for the left column, 1–4 for the right column). Axes bounds are common within columns

Figure 2 contains the traceplots of the MCMC estimation, ordered left to right, top to bottom, as reported in the model. Figure 3 contains the posterior densities for the hypotheses parameters *β* and the population means *α*. Most of the posterior distributions lie within the positive values of the support, with the exception of *β*_2_, which shows some mass (less than 10% of the posterior probability) in negative territory. Additionally, the need for construct-specific means is clear given the large differences across posterior densities of the parameters α and the construct-driven relationships. While Brand ethicality shows a large mean due to the lack of constructs influencing its variability, the other means are smaller and mostly driven by the level of contribution of the remaining constructs to their variability.

## Discussion

### Theoretical contributions

Kumar (2018) argues that customer behavior has shifted toward a need for authenticity, environmental awareness, and social connectivity. Brand ethics as an area of study is now more important than ever due to its impact on brand–customer interactions and the ensuing consumer behavior. This study builds on the model originally proposed by Iglesias et al. (2019), with four significant modifications from the references study: (1) it did not examine the moderating role of brand heritage on perceived brand ethicality; (2) instead of using brand equity as the dependent variable, we opted to explore the impact of perceived brand ethicality on consumer commitment towards the brand; (3) we examined the perception of brand ethicality in the highly relevant context of the COVID-19 pandemic, where brands were forced to operate in highly turbulent markets. Within this context, brands had to not only uphold their communicated brand ethical standards but also take advantage of the unique opportunity to promote trust, stability, and differentiation among internal and external stakeholders (Kay 2006). This can have a significant impact on brand commitment. As brand commitment can be considered to be an expression of the degree of devotion that a consumer feels towards a preferred brand (Barreda et al. 2020), brand commitment implies a concern from all parties engaged to develop an enduring relationship in the long term (Roberts et al. 2003), making it a critical target for organizations during turbulent market times; and (4) we examined brand ethicality in a general context without any particular business segment constraints, which made more sense given the context of the COVID-19 pandemic and the state of lockdown during which data were collected for this study as consumer needs and wants varied significantly.

Our results were in line with those presented in studies by Markovic et al. (2018) and Iglesias et al. (2019) under normal market conditions and pre-COVID-19 pandemic. The first hypothesis in our study examining the direct relationship between brand ethicality and brand commitment produced similar results to the previously mentioned studies, where the direct impact of brand ethicality on the examined variable resulted in the weakest association of all relationships examined. This suggests that brand perceived ethicality alone is insufficient to impact brand commitment during turbulent times meaningfully.

Hypotheses 2 and 5 examined the impact of brand ethicality on BRBs and brand image, respectively. The results here were similar to those presented in Iglesias et al. (2019), with a similar strength in the relationship of brand ethicality with the two constructs. Hypotheses 3 and 4 focused on the impact of BRBs in the model. We found that BRB has a strong relationship with brand image and with brand commitment. This is also in line with the results of Iglesias et al. (2019), and could be explained by the feeling of privilege that customers may experience when associating with a brand, as argued by Shugan (2005), which is impacted by the strong relationship with the ethical behavior of the brand in the context of this study.

Lastly, H6 displayed a significant relationship with brand commitment, though substantially smaller than BRB, signifying the stronger impact it has on brand commitment. In regards to brand image, it is interesting to note that brand ethicality had a similar effect (H5) as BRBs (H4). This supports the argument of Gürhan-Canli et al. (2016), regarding the growing importance of innovativeness, responsiveness, and responsibility as components of brand image, and how these components could become important concepts in a process of re-evaluation of the concept of brand image. The importance of these concepts might have been magnified by the COVID-19 scenario. Similar to Iglesias et al. (2019), H3 results also expressed a strong relationship between BRBs and brand commitment. While we anticipated that the relationships would be positive, as argued in the hypothesis, we did not expect BRB to exert such a strong impact on brand commitment given the context of the study focusing on tangible goods. The development of a positive brand image was found to be critical in the context of Iglesias et al. (2019) in which the focus was on service brands, where the intangibility of services and need for a strong brand image played a critical role. One possible explanation for our findings within tangible goods could be that a brand image can be considered a set of brand perceptions that results from the associations developed towards it that is held in the mind of individuals (Cretu and Brodie 2007). The relationship can be strongly affected by the benefits provided by the brand as an ethical brand, which may help support customers' self-identity and self-expression demands. This can be considered a concrete delivery of the promise made by the brand, but beyond that it can be considered to be the result of tangible and intangible brand associations (Barreda et al. 2020), with the intangibles in this case being represented by ethical behavior displayed by the brand. The new function of brands in a world that values their ethical behavior has substantial consequences for the development of social bonds. Most importantly, this new function raises concerns about the duties of “social brand engagement” (Kozinets 2014). Likewise, direct and indirect brand–customer interactions define the perception of a brand's image (Cho and Fiore 2015), thus justifying the similar degrees of impact that brand ethicality and BRBs have on brand image.

This study accentuates the need for investment in the development of favorable brand images for tangible goods through the adoption of robust brand ethicality programs. The perceived ethicality of a brand is an essential part of its image and reputation (Blombäck and Scandelius 2013). Our findings further reinforce the assertion in Iglesias et al. (2019) that ethics should be placed at the core of the brand from its genesis to set the baseline for the development of a positive brand image. The Bayesian approach in this manuscript provided full posterior information about existing relationships/links between the latent constructs (nodes in Fig. 1) from observations collected through multiple measurement items and treated as their noisy representations for the purposes of the Bayesian model. These representations take the form of a linear relationship, though the model can flexibly accommodate non-linear approaches when reasonable.

In addition to the aforementioned advantages, the Bayesian approach is not reliant on asymptotic theory. Large samples oftentimes come from large studies or long-time horizons. However, in many occasions the luxury of large datasets is not always available. For example, as structural shocks hit the markets, such as COVID-19, oftentimes quick responses are necessary, and collecting large sample sizes is in conflict with responsiveness. Low samples, as those more likely achieved during market shocks or structural shifts, are simply reflected into larger uncertainty about the parameters of interest. Also, since the Bayesian paradigm is parameter-centric, the aforementioned posterior distributions are direct representations of that uncertainty, as opposed to central-limit-theorem-based estimators. While we did not use prior information in this study, it could easily be incorporated, when available, through the use of more informative priors.

### Managerial implications, limitations and future research

The findings of this study can assist firms to better monitor consumer perception of brand features and behaviors resulting from brand ethics. The findings bolster the proposal by Ferrell et al. (2019) to continuously assess consumer impression of ethical activity. Positive brand attitudes and ethical actions can be identified, nurtured, and communicated to customers through this form of monitoring. Compliance strategies that increase brand attitude and financial value can help foster brand ethical conduct. While most previous studies on corporate ethics have focused on CSR, the findings in this article demonstrate that firms must also focus on maintaining positive ethical conducts beyond their CSR programs.

One of the contributions of this study is the evaluation of brand ethicality during turbulent times. Even though results showed that the direct impact of brand ethicality on brand commitment was moderate, the proposed model presented strong relationships with the other examined variables. BRBs and brand image were found to impact brand commitment strongly. In practical terms, this does not diminish the relevance of brand ethicality to support brand commitment. Instead, it means that management should still focus on it because of how it impacts BRBs and brand image, which in turn produced a strong relationship with brand commitment. In the case of the relationship between brand ethicality and BRB, consumers' strong perception of brand ethical behavior can lead to a more favorable BRB perception, further supporting consumer connection with the brand and ultimately leading to higher involvement and commitment. In the case of the relationship between brand ethicality and brand image, the strong evidence of the relationship suggests that it can help imbue the brand image with additional attributes valued by consumers resulting in a stronger brand image that has a favorable effect on consumer brand commitment. This suggests that the emotional advantages resulting from displays of brand ethical behavior represent a critical competitive advantage in establishing consumer brand commitment and merit further attention and action from management. The positive results found across hypothesized relationships indicate a strong synergy between all variables that can have a profound impact both on customers’ responses to ethical behavior and on their commitment towards a brand. The commitment to consumers expressed by values displayed through actions in a time of need is well-received by a brand’s customers, as indicated by more than half of the total population surveyed recently in an AMC Global study (Trentacosta 2020). Participants expressed the importance of hearing about specific actions that brands took to demonstrate their support to important causes. When asked specifically about ethical actions related to the COVID-19 pandemic, one consumer clearly stated that “Any company that has put out an advertisement regarding social injustice and COVID-19 is impressive to me because they are acknowledging that everyone deserves to be treated fairly.” The findings discussed in this paper are particularly important for brands currently operating in South America or considering expansion to the continent. The results provide strong evidence of the role ethical actions can have on consumer commitment towards brands.

This study is subject to a number of limitations that may be addressed in future research. However, it must be noted that despite that, it offers valuable theoretical and managerial contributions obtained at a time of high global volatility and uncertainty that put marketers to the test. First, in a perfect world, the data for this study would come from the same cross-section before and after the COVID pandemic. As this was not attainable, a point of comparison was determined based on prior studies examining brand ethicality by Markovic et al. (2018) and Iglesias et al. (2019). While not ideal, at least a comparison with similar studies can serve as a point of reference to contrast results. Second, there is the issue of mono-method bias, as all data used in the analysis is acquired via surveys. Given the timeframe and the need to capture data at a critical moment of the pandemic a more representative sample of the population could not be captured. Additionally, the limitations embedded in the sampling approach used for this study are quite obvious as discussed in the methodology section, however, it allows for a speedy data collection process which was of the essence for this study. The population captured in the sample skewed toward those in higher education, and, therefore, cannot be said to be representative of the overall population, even if those passed it along to others. Future studies could be enhanced through mixed methods approaches to gain additional insights about customers’ opinions of brand ethicalness and to uncover behavioral repercussions. Third, the sample only includes Colombians and legal residents of the country who self-identified when completing the survey, therefore the conclusions cannot be generalized. Additionally, a large portion of the population was removed from the study due to the lack of access to a computer to fill the survey or even the ability to read and comprehend the questions, as is the reality in emerging countries. It would be interesting to examine brand ethicality perceptions of that portion of the population, who also consumes goods, and would probably be poorer and have different perceptions of branding. While this study represents a pilot during a key time point in the pandemic, future research could address this problem by replicating this study in other nations facing similar stress scenarios. Studies in other countries could incorporate relevant cultural variables that may influence brand ethicality judgments on various outcomes. Because of the global interconnectedness, the COVID-19 pandemic has highlighted the relevance of an international viewpoint in marketing literature development. While regional research might be valuable, a global perspective should be pursued whenever possible. In this study, lastly, while this study focuses on brands, similar studies focused on how front-line personnel might affect the growth of customers' perceptions of brand ethicality across numerous touch points in the customer journey would be worthwhile. This is particularly relevant in this context due to the role that delivery services have played in satisfying consumers’ needs during the pandemic.

Predicting what the post-COVID-19 marketing strategy world will entail when exploring turbulent conditions is difficult. According to He et al. (2012), organizational goals may change over time, necessitating revision of vision statements. While organizations preparing for a post-pandemic world will need to re-evaluate their visions, missions, and objectives to account for changes in their customers and competitors, consumers will continue to have extremely high expectations of brands' ethical behavior, if not increased, in light of how brands responded to the COVID-19 pandemic.

## Appendix: Description of the model

We denote each of the individuals in the sample with sub-indices *i* = 1,..,*n* = 761. Index *j* corresponds to each of the Likert-based questions across each of the latent constructs/brand characteristics *k* = *4* (*j* = 1,…,*J*(*k*)). The *k* = 1,..,4 index represents Brand Ethicality, Brand Benefits, Brand Commitment and Brand Image, respectively. Index *s* represents each of the Likert categories (*s* = 1,..,*S* = 7) for each of the items (all items were represented under a 7-level Likert scale). The observed responses to each of the items are represented as *Y*_*i,j,k*_ = *s*, with sub-indices representing the respondent (*i*), question identifier within the construct (*j*), and construct (*k*).

Latent probabilities of each of the Likert items are represented as the (*n* by *s* by *k*) tensor *p*, and the latent constructs are represented by *Z*_*i,k*_ for each individual and brand characteristic pair, with corresponding latent mean matrix *μ*_*i,k*_. Latent hyper-parameters linking the constructs are represented as *α* (population levels) and *β* (linear relationships), with the latter representing the underlying hypotheses of our study. Hidden thresholds in the multinomial representation of the problem are represented as *τ* (*k* by *S* − 1 matrix, with the first column set to zero for identifiability). For a complete description of these types of models, see Johnson and Albert (2006).

To complete the notation, let Φ denote the Standard Normal cumulative density function; MN(*p*) denote the multinomial probability mass function; *N*(*m*, *s*) denote a normal probability density function with *m* = mean and *s* = standard deviation; and Ga(*α*, *β*) denote the Gamma probability density function with parameters *α* and *β*.

The model details can be summarized as follows:

$${Y}_{i,j,k} \sim \mathrm{MN}\left({p}_{{\varvec{i}},{\varvec{k}}}\right),$$

$${p}_{i,k,1}=\Phi \left(-{Z}_{i,k}\right),$$

$${p}_{i,k,s}=\Phi \left({\tau }_{k,s}-{Z}_{i,k}\right) - \Phi \left({\tau }_{k,s-1}-{Z}_{i,k}\right) s=2,\dots ,6,$$

$${p}_{i,k,7}=1- \sum_{s=1}^{6}{p}_{i,k,s},$$

$${Z}_{i,k}=N\left({\mu }_{i,k},1\right),$$

$${\mu }_{i,1}={\alpha }_{1} ,$$

$${\mu }_{i,2}={\alpha }_{2}+{\beta }_{1}{Z}_{i,1},$$

$${\mu }_{i,3}={\alpha }_{3} +{\beta }_{2}{Z}_{i,1}+{\beta }_{3}{Z}_{i,2} +{\beta }_{4}{Z}_{i,4},$$

$${\mu }_{i,4}={\alpha }_{4} +{\beta }_{5}{Z}_{i,1}+{\beta }_{6}{Z}_{i,2} ,$$

$${\beta }_{k},{\alpha }_{k} \sim N\left(0,s\right),$$

$${\tau }_{i,1}\equiv 0,$$

$${\tau }_{k,s}=\sum_{i=1}^{s-1}{g}_{k,s} s=2,\dots ,6,$$

$${g}_{k,s} \sim \mathrm{Ga}\left(a,b\right).$$

The intuition behind the model can be described through the hierarchical levels:

1. It is assumed that the observed Likert ordinal response tensor *Y* is driven by a multinomial distribution for each item, with probability vector that is individual- and construct-specific. This allows for heterogeneity within the responses, where those are different across individuals and constructs.
2. The probability vector is mapped through an ordinal Probit model into areas in the real line, with the surface of that area driven by individual-specific means. We assume that the true opinions across individuals and constructs (matrix *Z*) are latent and unknown, and they are observed with variability across the construct-specific items.
3. The hypotheses relate the constructs between themselves through linear relationships *β* after accounting for the population mean levels *α* for each construct.
4. The final layer represents the hyper-priors, which are assumed non-informative, since we do not have prior studies on which to build any knowledge about the relationships

The model was run for 100,000 iterations using OPENBUGS, with a burn-in of 10,000 iterations. Hyper-prior parameters were set to 0.001. A sensitivity analysis was performed with other parameter values with no discernible impact on the posterior distribution. The results shown in Table 3 correspond to the summary of the posterior distribution for the hypotheses-specific parameters.

There are minor differences between the ordered Probit and other ordered categorical models. However, this type of models allows for differences between scales to be flexibly mapped to differences in the latent construct. For example, when coding a 5-scale Likert variable with categories very good, good, neutral, bad, and very bad, there is no guarantee that the respondent considers the distance between very good and good as the same distance between good and neutral. An ordered categorical model estimates the thresholds between these scales, rather than imposing equi-distant thresholds, hence offering protection against large departures from equidistance assumptions, and flexibly estimating thresholds between categories.
